# Leveraging Biomarkers of Aging

**DOI:** 10.1093/geroni/igaf122.1667

**Published:** 2025-12-31

**Authors:** Nathan LeBrasseur

## Abstract

There is considerable interest in using diverse forms of data—e.g., routine and advanced laboratory tests, diagnostic images, physiological measures, and wearable outputs—to assess states of biological vs. chronological age and rates of biological vs. chronological aging. Without question, biomarkers offer incredible potential in research and clinical practice as predictors of future health and as informers of what, how, when, and in whom exposures, behaviors, and interventions affect aging. This symposium will provide an update on the latest advances in, and strengths and weaknesses of, biomarkers of aging and their application.

